# Immunogenicity and Protective Activity of a Chimeric Protein Based on the Domain III of the Tick-Borne Encephalitis Virus E Protein and the OmpF Porin of *Yersinia pseudotuberculosis* Incorporated into the TI-Complex

**DOI:** 10.3390/ijms19102988

**Published:** 2018-09-30

**Authors:** Nina Sanina, Natalia Chopenko, Andrey Mazeika, Ludmila Davydova, Galina Leonova, Anna Stenkova, Vladimir N. Uversky, Eduard Kostetsky

**Affiliations:** 1 Far Eastern Federal University, 8 Sukhanova St., Vladivostok 690091, Russia; natali_1389@mail.ru (N.C.); nikandrei@inbox.ru (A.M.); l.vorobushek@mail.ru (L.D.); stenkova@gmail.com (A.S.); kostetskiy.yeya@dvfu.ru (E.K.); 2 Somov Institute of Epidemiology and Microbiology, 1 Selskaya st., Vladivostok 690087, Russia; galinaleon41@gmail.com; 3 G.B. Elyakov Pacific Institute of Bioorganic Chemistry, FEB RAS, Prospect 100 let Vladivostoku, 159, Vladivostok 690022, Russia; 4 Department of Molecular Medicine and USF Health Byrd Alzheimer’s Research Institute, Morsani College of Medicine, University of South Florida, 12901 Bruce B. Downs Blvd. MDC07, Tampa, FL 33612, USA; vuversky@health.usf.edu; 5 Laboratory of New methods in Biology, Institute for Biological Instrumentation, Russian Academy of Sciences, Pushchino 142290, Moscow Region, Russia

**Keywords:** nanoparticulate adjuvant, nanoparticulate delivery system, fusion antigen, subunit vaccines, monogalactosyldiacylglycerol, TBEV

## Abstract

Tick-borne encephalitis (TBE) is a widespread, dangerous infection. Unfortunately, all attempts to create safe anti-TBE subunit vaccines are still unsuccessful due to their low immunogenicity. The goal of the present work was to investigate the immunogenicity of a recombinant chimeric protein created by the fusion of the EIII protein, comprising domain III and a stem region of the tick-borne encephalitis virus (TBEV) E protein, and the OmpF porin of *Yersinia pseudotuberculosis* (OmpF-EIII). Adjuvanted antigen delivery systems, the tubular immunostimulating complexes (TI-complexes) based on the monogalactosyldiacylglycerol from different marine macrophytes, were used to enhance the immunogenicity of OmpF-EIII. Also, the chimeric protein incorporated into the most effective TI-complex was used to study its protective activity. The content of anti-OmpF-EIII antibodies was estimated in mice blood serum by enzyme-linked immunosorbent assay (ELISA). To study protective activity, previously immunized mice were infected with TBEV strain Dal’negorsk (GenBank ID: FJ402886). The animal survival was monitored daily for 21 days. OmpF-EIII incorporated into the TI-complexes induced about a 30–60- and 5–10-fold increase in the production of anti-OmpF-EIII and anti-EIII antibodies, respectively, in comparison with the effect of an individual OmpF-EIII. The most effective vaccine construction provided 60% protection. Despite the dramatic effect on the specific antibody titer, the studied TI-complex did not provide a statistically significant increase in the protection of OmpF-EIII protein. However, our results provide the basis of the future search for approaches to design and optimize the anti-TBEV vaccine based on the OmpF-EIII protein.

## 1. Introduction

Tick-borne encephalitis (TBE) is a widespread infection of the central nervous system, which is induced by the tick-borne encephalitis virus (TBEV) belonging to the genus *Flavivirus* of the Flaviviridae family. More than 80 years have passed since the discovery of the causative agent of TBE, which can lead to neurologic complications and death [1]. However, this dangerous neuroinfectious disease has not yet been liquidated. On the contrary, the incidence rate is increasing, and new foci have appeared [2].

The most effective way to fight TBE is vaccination. Despite the high efficiency of the currently used inactivated TBE vaccines, their production is associated with processing of a large number of dangerous pathogens. Therefore, the development of new vaccines with a safe production process that could cause prolonged immunity without additional revaccinations is needed [3]. In connection with this need, the current trend is the creation of safe subunit vaccines, which contain only the part of the pathogen (antigen) that is responsible for producing anti-infectious immunity in animals or humans. Most protective antigens of microorganisms are membrane proteins, which can be obtained using recombinant strategies. Recombinant protein antigens, unlike their analogues isolated from pathogens, have a clear advantage in terms of the safety and reproducibility of the procedures for obtaining the antigens, as well as improving the environmental situation associated with the vaccine manufacturing. The use of purposefully constructed recombinant chimeric proteins with given properties is a new promising approach to the creation of subunit vaccines. However, such antigens are generally poorly immunogenic, and need specific adjuvants [4]. Only a few of them are suitable for medical and veterinary vaccines in spite of a wide arsenal of available adjuvants [5,6].

The envelope (E) protein of TBEV contains the antigenic determinants responsible for haemagglutination and neutralization, and induces protective immunity in the host [7]. The E protein contains 3 domains. The domain III (DIII) of E protein is the main antigenic domain, which includes virus-specific epitopes recognized by neutralizing antibodies [8,9]. Therefore, we have constructed the chimeric protein OmpF-EIII based on the fusion of EIII, which consists of DIII and a stem of E protein, and porin OmpF of Gram-negative bacteria *Yersinia pseudotuberculosis*, for the further development of a subunit anti-TBE vaccine [10]. Membrane protein OmpF is necessary as a hydrophobic anchor, which allows incorporation of the fusion antigen into the lipid matrix of the nanoparticulate adjuvant and the antigen delivery system, such as the tubular immunostimulating complexes (TI-complexes) [5,11]. OmpF is also required to present the recombinant antigens in the optimal form to the immunocompetent cells. TI-complexes are virus-like nanoparticles consisting of cholesterol, saponin cucumarioside A_2_-2 (CDA), and glycolipid monogalactosyldiacylglycerol (MGDG) isolated from marine macrophytes. To regulate the conformation of hybrid antigens in TI-complexes, and to obtain the most immunogenic vaccine construction, MGDGs with different physicochemical properties were used, since MGDG forms a lipid matrix for the antigen incorporated in the TI-complex.

The aim of the present work was to study the immunogenicity of the chimeric protein OmpF-EIII incorporated into TI-complexes containing MGDGs isolated from different marine macrophytes (algae and seagrass), and therefore characterized by different fatty acid compositions [12,13] and microviscosity [14]. The other aim was to determine the anti-TBEV protective activity of the OmpF-EIII incorporated in the most effective TI-complex.

## 2. Results

### 2.1. Choice of the OmpF-EIII Antigen Dose and Scheme of Immunization

**Figure 1:**
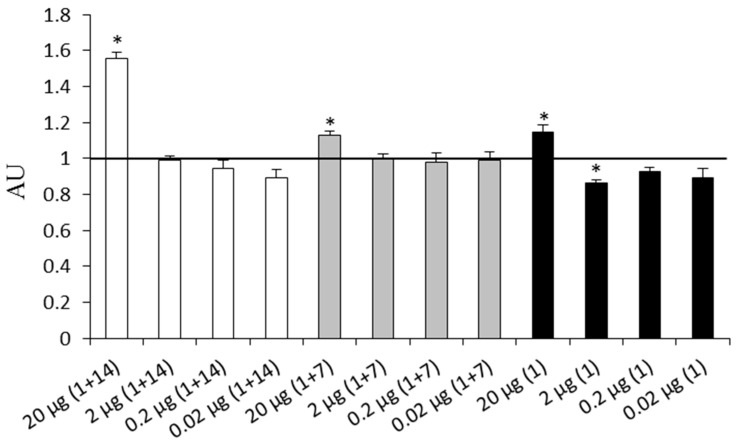
Effects of different doses of OmpF-EIII and different schemes of immunization on the content of anti-OmpF-EIII antibodies in mice blood sera. *X*-axis: Experimental groups of animals injected subcutaneously once (1), or twice at an interval of 7 days (1 + 7) or 14 days (1 + 14), with OmpF-EIII at doses of 20, 2, 0.2, or 0.02 μg/mouse. The experiment was terminated 28 days after the first immunization. Each experimental group included ten mice. The control group included mice injected with phosphate-buffered saline (PBS) containing 0.125% *n*-octylglucoside. The content of antibodies was evaluated by enzyme-linked immunosorbent assay (ELISA), and expressed as the ratio between the absorption in experimental groups and the control value equal to 1 (the horizontal line) (*Y*-axis). Data are presented in arbitrary units (AU). The dilution of mouse blood serum was 1/800. Bars represent mean ± SE. * *p* < 0.05 as compared with the control.

Figure 1 shows the results of mice immunization with the OmpF-EIII protein at different doses (0.02, 0.2, 2.0, and 20 μg/mouse) that allowed determination of the optimal protein dose of 20 μg/mouse. The effects of the antigen doses of 20 and 40 μg/mouse were the same. The test of three schemes of immunization also demonstrated that only the immunization scheme consisting of two immunizations at an interval of 14 days (1 + 14) resulted in the enhancement of the anti-OmpF-EIII antibodies level in comparison with the control. Other schemes (single immunization (1) and two immunizations at an interval of seven days (1 + 7)) were ineffective. Therefore, two immunizations at an interval of 14 days (1 + 14) with the OmpF-EIII protein at a dose of 20 μg/mouse was used in the following study of OmpF-EIII protective activity.

### 2.2. Adjuvant Effect of TI-Complexes on Immunogenicity of OmpF-EIII

**Figure 2:**
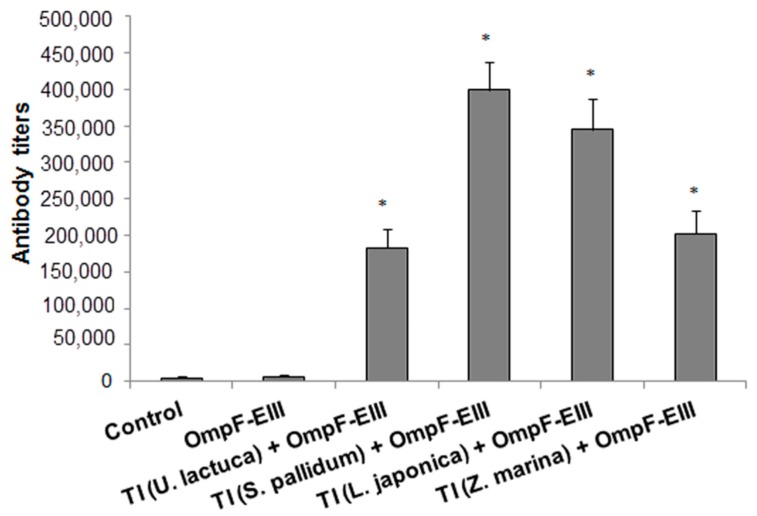
The content of the anti-OmpF-EIII antibodies in mice blood sera depending on the monogalactosyldiacylglycerol (MGDG) in the composition of the TI-complexes. Antibodies were evaluated by ELISA in mice blood sera after two immunizations with individual EIII-OmpF (OmpF-EIII) and OmpF-EIII incorporated in TI-complexes based on the MGDG from *Ulva lactuca* (TI (*U. lactuca* + OmpF-EIII)), *Sargassum pallidum* (TI (*S. pallidum* + OmpF-EIII)), *Laminaria japonica* (TI (*L. japonica* + OmpF-EIII)), or *Zostera marina* (TI (*Z. marina* + OmpF-EIII)), respectively, at a dose of 20 μg/mouse, administered subcutaneously at an interval of 14 days. The control group included mice immunized with PBS containing 0.125% *n*-octylglucoside. Each experimental group included ten mice. The experiment was terminated 28 days after the first immunization. *Y*-axis: The content of anti-OmpF-EIII antibodies in the experimental groups. Data are expressed in titers. Bars represent mean ± SE. * *p* < 0.05 as compared with the OmpF-EIII.

Since the fatty acid composition and microviscosity of the glycolipid constituent of TI-complexes can significantly affect the conformation and immunogenicity of a protein antigen [14,15,16], OmpF-EIII was incorporated into the TI-complexes containing MGDG isolated from different marine macrophytes [11,12]. As shown in Figure 2, the production of anti-OmpF-EIII antibodies increased by 29–63 times in the groups of mice immunized with OmpF-EIII incorporated in TI-complexes based on MGDG from marine macroalgae *Laminaria japonica*, *Sargassum pallidum*, *Ulva lactuca*, and seagrass *Zostera marina*, in comparison with the group of mice immunized with the individual protein antigen. TI-complexes based on the MGDG from *S. pallidum* and *L. japonica* induced maximal adjuvant effect, where the effect of the TI-complex based on the MGDG from *S. pallidum* was more pronounced. In turn, the efficiency of TI-complexes based on the MGDGs from *U. lactuca* and *Z. marina* was the lowest, which may be due to the very different fatty acid compositions of the MGDG from these macrophytes (characterized by the much higher values of the ratios between the unsaturated and saturated fatty acids, and between *n*−3 and *n−*6 polyunsaturated fatty acids due to the accumulation of 16:4*n−*3 or 18:3*n*−3 fatty acids) [11].

**Figure 3:**
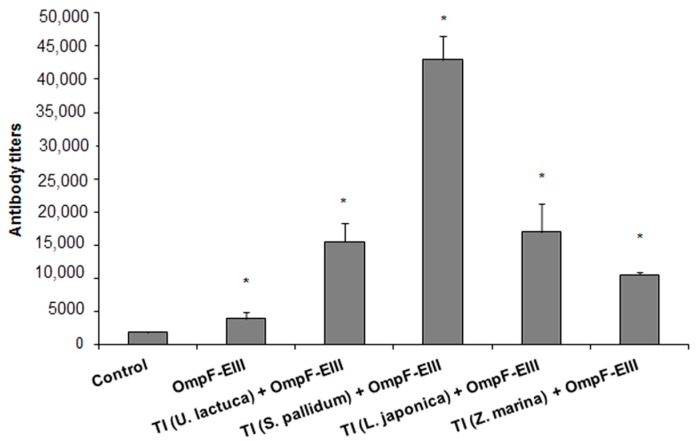
The content of anti-EIII antibodies in mice blood sera depending on the MGDG constituent of TI-complexes. Antibodies were evaluated by ELISA in mice blood sera after two immunizations with individual EIII-OmpF (OmpF-EIII) and OmpF-EIII incorporated in TI-complexes based on MGDG from *Ulva lactuca* (TI (*U. lactuca* + OmpF-EIII)), *Sargassum pallidum* (TI (*S. pallidum* + OmpF-EIII)), *Laminaria japonica* (TI (*L. japonica* + OmpF-EIII)), or *Zostera marina* (TI (*Z. marina* + OmpF-EIII)), respectively, at a dose of 20 μg/mouse, administered subcutaneously at an interval of 14 days. The control group included mice immunized with PBS containing 0.125% *n*-octylglucoside. Each experimental group included ten mice. The experiment was terminated 28 days after the first immunization. *Y*-axis: The content of anti-EIII antibodies in the experimental groups. Data are expressed in titers. Bars represent mean ± SE. * *p* < 0.05 as compared with the OmpF-EIII.

The clear advantage of the TI-complex based on MGDG isolated from *S. pallidum* is further illustrated by Figure 3, demonstrating the dependence of the anti-EIII antibody level on the glycolipid constituent in the composition of TI-complexes. The EIII-OmpF protein incorporated into this TI-complex induced a 24-fold increase in the anti-EIII antibody level in comparison with the control value. Other TI-complexes promoted a 6–9-fold increase, and therefore were much less effective. In turn, adjuvant properties of all studied TI-complexes resulted in a significant increase in the stimulating effect of the EIII-OmpF protein, which being injected alone, induced a 2-fold increase in the anti-EIII antibody level in comparison with the control value. 

The 10-fold increase in the antibody titer by day 28 after the first immunization in comparison with the initial levels was detected in 80% and 50% of the animals immunized with the chimeric protein OmpF-EIII incorporated in TI-complexes based on MGDG from *S. pallidum* and *U. lactuca*/*L. japonica*, respectively. However, seroconversion defined as a 10-fold rise in antibody titer was not observed in the groups of animals injected with the individual chimeric protein and chimeric protein incorporated in the TI-complex based on MGDG from *Z. marina*. Therefore, these results indicated choosing the TI-complex based on MGDG from *S. pallidum* as the most effective system for the following experiments on the protective activity of OmpF-EIII. 

### 2.3. Protective Activity of OmpF-EIII Alone and OmpF-EIII Incorporated into the TI-Complex

**Figure 4:**
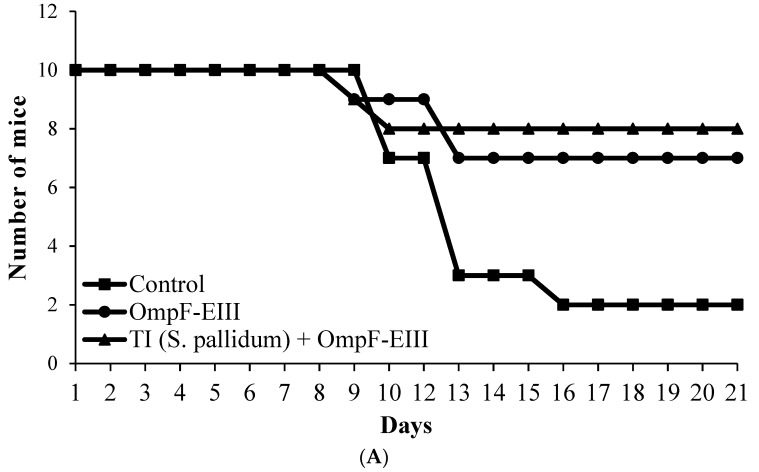

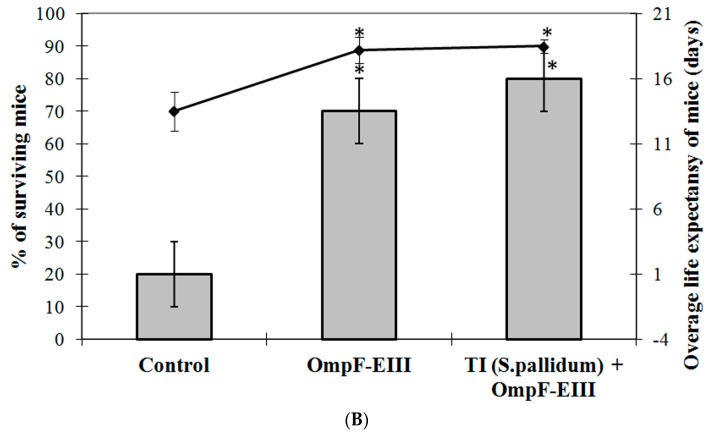
Protective activity of OmpF-EIII alone and OmpF-EIII incorporated into the TI-complex (TI (*S. pallidum*) + OmpF-EIII). (**A**) Number of mice surviving post infection with the tick-born encephalitis virus (TBEV) in groups of animals previously immunized twice with the OmpF-EIII alone (OmpF-EIII) and OmpF-EIII incorporated into the TI complex (OmpF-EIII + TI). Control represents a group of animals infected with TBEV without previous immunization. Each group contained ten animals. The duration of observation was 21 days post infection. (**B**) Percentage of survival (bars) in groups of animals: Control, OmpF-EIII, and TI (*S. pallidum*) + OmpF-EIII. Average life expectancy (line graph) in groups of animals: Control, OmpF-EIII, and TI (*S. pallidum*) + OmpF-EIII. Values are means ± SE for three independent experiments. The Fisher exact test was used to compare the protective activity in mice groups with the control. * *p* < 0.05 as compared with the control.

The dynamics of the change in the number of animals that survived after TBE infection is shown in Figure 4A. In the group of non-immunized mice, only 2 out of 10 animals survived at the end of the experiment, whereas 7 animals survived in the group of mice immunized with the OmpF-EIII protein alone. The incorporation of the chimeric protein into the TI-complex based on the MGDG from *S. pallidum* further increased the number of surviving animals to eight. With this, the average life expectancy increased from 13.5 days in the control group to 18.2 days and 18.5 days in groups of mice immunized with the chimeric protein alone and the OmpF-EIII protein incorporated into the TI-complex, respectively (Figure 4B). Therefore, the percentage of surviving mice increased from 20% in the control group to 70% and 80% in groups of mice immunized with OmpF-EIII alone and OmpF-EIII incorporated in the TI-complex, respectively. In other words, the level of protection against the TBE infection reached 60% in the group of mice immunized with the chimeric protein incorporated into the TI-complex based on the MGDG from *S. pallidum* (Figure 4B). However, the 10% increase in the level of protection due to the incorporation of the OmpF-EIII protein into TI complexes was not statistically significant.

### 2.4. Evaluation of the Intrinsic Disorder Predisposition of OmpF-EIII

To provide some clues to the structural basis of the efficient immunogenicity of the OmpF-EIII, we evaluated the intrinsic disorder predisposition of this chimeric protein using a comprehensive computational approach based on the utilization of several tools designed for characterization of various flavors of intrinsic disorder. As described earlier [10], the amino acid sequence of the chimeric OmpF-EIII protein includes 499 residues: 

MHHHHHHAEIYNKDGNKLDLYGKVDARHSFSDNNKQDGDKSYVRFGFKGETQITDQLTGYGQWEYNIQANNAEDSGAQDGNATRLGFAGLKFAEFGSFDYGRNYGVIYDVNAWTDMLPVFGGDSISNSDNFMTGRSTGLATYRNNNFFGMVDGLNFALQYQGKNDRSEVKEANGDGFGIGSTYDLGNGINFGAGFSSSNRTLDQKYGSTAEGDKAQAWNVGAKYDANNVYLAVMYAETQNLTPYGFYDFTIANKTRDIEITAQYQFDFGLRPSLGYVQSKGKDLNNVDANHDLVKYVSVGTYYYFNKNMSTYVDYKINLLDKDLFTEVNRIMTDDVVAVGLVYQFGDGAGLINGLTYTMCDKTKFTWKRIPTDSGHDTVVMEVAFSGTKPCRIPVRAVAHGSPDVNVAMLITPNPTIENNGGGFIEMQLPPGDNIIYVGELSHQWFQKGSSIGRVFQKTRKGIERLTVIGEHAWDFGSTGGFLASVGKALHTVLGGAFN

where regions of 6× His tag, OmpF of *Y. pseudotuberculosis*, linker, the DIII domain, and a stem of the TBEV E protein are shown in brown, black, red, green, and violet fonts, respectively.

**Figure 5:**
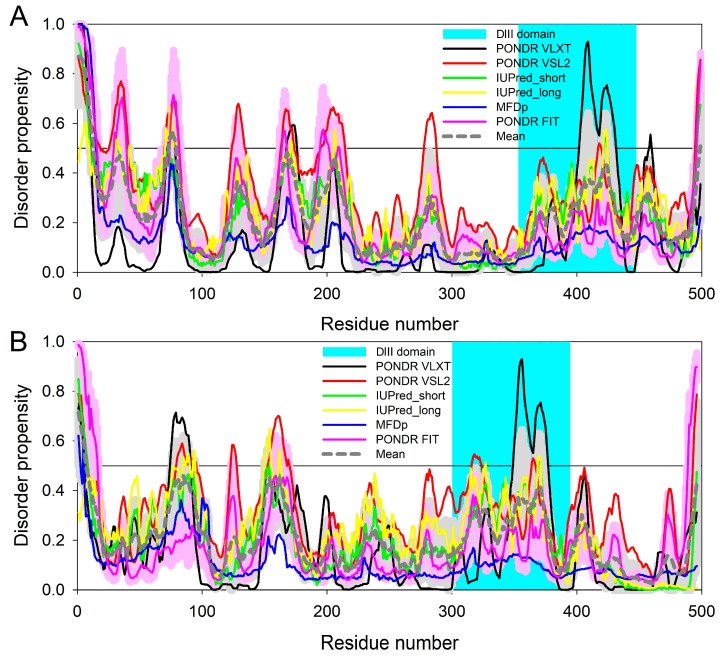
Intrinsic disorder predisposition of the chimeric OmpF-EIII protein (**A**) and the mature tick-borne encephalitis virus E protein (**B**) evaluated by several common per-residue predictors of intrinsic disorder, such as PONDR^®^ VLXT (black line), PONDR^®^ VSL2 (red line), IUPred_short (green line), IUPred_long (yellow line), MFDp (blue line), and PONDR^®^ FIT (pink line). These computational tools were selected because of their different sensitivity to different disorder-related features. The bold, dashed dark gray line shows the mean disorder propensity calculated by averaging the disorder profiles of individual predictors. The light pink shadow around the PONDR^®^ FIT curve shows the error distribution for this predictor, whereas the light gray shadow around the mean disorder propensity curve represents the distribution of standard deviations for this tool. The position of the DIII domain of the tick-borne encephalitis virus E protein (residues 354–447 of the chimeric protein) is shown by the light cyan bar. The most C-terminal part (residues 448–499 of the chimeric protein) corresponds to a stem of the E protein. In these analyses, the predicted intrinsic disorder scores above 0.5 are considered to correspond to the disordered residues or regions, whereas residues and regions with the disorder scores >0.2 are considered as flexible.

The results of this comprehensive computational analysis are shown in Figure 5A, which represents a set of resulting disorder profiles of the chimeric OmpF-EIII protein. It is clear that the DIII domain of the tick-borne encephalitis virus E protein, serving as the main antigenic domain that contains virus-specific epitopes recognized by neutralizing antibodies [8,9], is characterized by noticeable conformational flexibility. 

It is of interest that despite their similar size (499 residues in OmpF-EIII vs. 498 residues in protein E), and despite the fact that both the chimeric OmpF-EIII protein and mature viral E protein are transmembrane proteins, disorder profiles of these two proteins in the vicinity of the DIII domain, especially at its N-terminal part, are rather different (cf. plots A and B in Figure 5). Furthermore, although the native protein E is anchored to the viral membrane via its C-terminal most anchor region located at the C-tail of the DIV domain (the stem + the hydrophobic anchor), the chimeric OmpF-EIII protein is embedded to the membrane via the OmpF protein (which is a channel-forming 16-stranded β-barrel) located before the DIII domain. Although in the protein E, domain DIII is known to be engaged in interaction with domains DI and DII, forming together with the domain DI a pocket that covers a fusion loop of the domain DII, it is likely that in the OmpF-EIII protein, the DIII domain represents a standalone structure protruding from the membrane. This positioning of the DIII domain within the chimeric protein and the intrinsic flexibility of this domain makes it easily accessible to the immune system.

## 3. Discussion

Currently, recombinant strategies are dominant in the elaboration of modern subunit vaccines, primarily due to the safety and reproducibility of the procedures utilized for obtaining recombinant proteins in comparison with the procedures used in obtaining proteins directly from the pathogens. Therefore, the problem of producing preparative quantities of various recombinant proteins of pathogens is extremely relevant. Despite the obvious advantages over widely used classic vaccines, recombinant subunit vaccines are not efficient without the use of specific adjuvants, antigen delivery systems, or both. Therefore, there is a clear need for development of new and improved adjuvants for the development of vaccines, in particular those that are based on the highly pure, but poorly immunogenic recombinant proteins. Among antigen delivery systems, micro- and nano-particles are the most attractive candidates, because they can enhance the cross-presentation of the antigen, and activate both innate and adaptive immune systems [17]. Virus-like immune stimulating complexes (ISCOMs) are considered as “gold standard” delivery systems, which dramatically exceed liposomes and aluminum adjuvants in efficiency, stability, and other characteristics. Nevertheless, fundamentally new in morphology and composition, the TI-complexes demonstrated greater adjuvant efficiency in relation to OmpF porin isolated from *Y. pseudotuberculosis* in comparison with the ISCOM and Freund’s complete adjuvant [11]. The efficiency of TI-complexes was shown to depend on the MGDG constituent that surrounds the incorporated antigen and influences its conformation [14]. This mechanism allows regulation of immune response against the incorporated antigen, and optimizes vaccine constructions, based on the composition of the TI-complexes. 

The present work confirmed our conclusions about the effect of TI-complexes on the immunogenicity of subunit antigens (recombinant and isolated ones), which were made based on earlier data. Compared to the individual OmpF-EIII protein, the OmpF-EIII protein incorporated into TI-complexes significantly stimulated the production of antibodies against OmpF-EIII. Furthermore, the immunogenicity of OmpF-EIII was dramatically increased under the influence of TI-complexes in comparison with the earlier studied immunogenicity of a recombinant monomer of the influenza virus hemagglutinin [15] and its subunit HA1 [16], as well as that of OmpF isolated from *Y. pseudotuberculosis* [14] incorporated into TI-complexes. The adjuvant effects of the TI-complexes on OmpF-EIII depended on the nature of the MGDG constituent, as was noted for the OmpF isolated from *Y. pseudotuberculosis* [14] and the recombinant subunit HA1 of the influenza virus hemagglutinin [16]. Similar to the data of our previous studies, the results of the present work showed that the TI-complex based on the MGDG from *S. pallidum* was characterized by the highest adjuvant activity. The medium microviscosity of this MGDG sample probably also promotes moderate changes in the OmpF-EIII conformation and the optimal presentation of this antigen to the immune system, as was earlier shown for OmpF isolated from *Y. pseudotuberculosis* and the recombinant subunit HA1 of the influenza virus hemagglutinin. The TI-complex based on the MGDG from *S. pallidum* provided the most efficient increase in the levels of the anti-OmpF-EIII, and especially the anti-EIII antibodies. Therefore, this TI-complex was chosen for the subsequent analysis of the protective activity. The experiments on the protective activity have shown that individual chimeric protein OmpF-EIII possessed high protective potential on its own (protection of 50%). However, the incorporation of OmpF-EIII into the TI-complex based on the MGDG from *S. pallidum* provided a protection of 60% in comparison with the control. Therefore, the protection of individual protein OmpF-EIII alone, and this protein incorporated into the TI-complex, was approximately equal despite the dramatic difference in the anti-EIII antibodies titer. Perhaps the 10-fold increase in the anti-EIII antibodies titer due to the incorporation of the protein antigen into TI-complex was accompanied by a decrease in the affinity of the antibodies, similar to the known effect of increasing the dose of antigen. Also, the possible cause may by a greater shift of the Th1/Th2 balance toward Th2 [18,19] under the influence of the OmpF-EIII protein incorporated into the TI-complex, since a decrease in the number of the Th1-lymphocyte subpopulation makes it difficult to eliminate the intracellular pathogen, and reduces the effect of antiviral protection. 

Nevertheless, the protection of the OmpF-EIII protein incorporated into the TI-complex is 10% higher in comparison with the result described in [3], where chimeric proteins based on the fusion of three variants of recombinant TBEV E protein domain III of European, Siberian, and Far Eastern subtypes, with dextran-binding domain of *Leuconostoc citreum* KM20 immobilized on a dextran carrier with CpG oligonucleotides as an adjuvant, have shown 50% protection against the Siberian strain of TBEV. Unfortunately, the protective activity of this construction against the Far Eastern strain, and the contribution of the chimeric protein alone, were not analyzed in that study. Unlike the TI-complexes, the dextran carrier with CpG oligonucleotides does not allow regulation of the conformation of a recombinant antigen, and thereby is poorly suited for the optimization of the vaccine construction. The increased conformational flexibility of the DIII domain in the chimeric protein OmpF-EIII, as compared to its flexibility within the TBEV E protein demonstrated by computational analysis, represents an important factor allowing this region to change its spatial structure in response to changes in the lipid surroundings of OmpF-EIII at incorporation into the TI-complexes, and thereby optimally represent the main virus-specific epitopes to the immune system. The results obtained in our study provide the basis for further research into approaches to design and optimize the subunit-based vaccines against TBEV.

## 4. Materials and Methods

### 4.1. Chimeric Protein Antigen

The construction, expression, and purification of recombinant OmpF-EIII antigen was carried out as previously described [10]. The presence of proteins throughout the purification was monitored by SDS-PAGE according to Laemmli using 12% polyacrylamide gel [20]. The antigen was detected by immunoblotting, as previously described [10].

### 4.2. Preparation of MGDG

The MGDG samples were isolated from four species of marine macrophytes: *L. japonica*, *S. pallidum* (Phaeophyta), *U. lactuca* (Chlorophyta), and *Z. marina* (Embryophyta). These marine macrophytes from the Sea of Japan were collected in summer, when the seawater temperature was 20–23 °C. To inactivate algae and seagrass enzymes, freshly harvested samples of these macrophytes were submerged in boiling water for 2 min. Resulting preparations of marine macrophytes were used to isolate MGDG samples according to previously described protocols [12].

### 4.3. Preparation of the OmpF-EIII-Containing TI-Complexes

TI-complexes were prepared as previously described [11]. Here, the stock solutions of MGDG, cholesterol, and CDA isolated from the marine invertebrate *Cucumaria japonica* according to [21] were prepared by dissolving 5 mg of MGDG or 5 mg of cholesterol in 1 mL of chloroform, and by dissolving 4 mg of CDA in 1 mL of distilled water. The 132 and 66 μL aliquotes of the MGDG and cholesterol solutions, respectively, were evaporated to dryness under a stream of air at a temperature of 60 °C, and the resulting dry residue was mixed with 84 μL of the CDA solution. The concentration of MGDG and cholesterol was adjusted to 2 mg/mL by adding 416 μL of phosphate-buffered saline (PBS) (pH 7.2) to the resulting mixture. A SONOPULS HD 2070 (Bandelin, Berlin, Germany) ultrasonic disintegrator was used for sonication of the resulting suspension for 5 min at 10% of maximum power, in the mode of 0.7 s—work; 0.3 s—interval. These sonicated preparations were stored for less than 24 h at 4 °C.

The TI-complex containing 20 μg of the OmpF-EIII protein per 10 μg of CDA was generated by mixing of 165 μL of the freshly sonicated TI-complex preparation with 220 μL of the OmpF-EIII protein solution in PBS (pH 7.2) at a concentration of 1 mg/mL. The final volume of the sample was brought to 1000 μL, and the resulting mixture was vortexed for 1 min. These samples were stored for less than 24 h at 4 °C.

To obtain the TI-complexes containing 2, 0.2, and 0.02 μg of the OmpF-EIII protein per 10 μg of CDA, 22 and 2.2 μL of the OmpF-EIII protein solution at a concentration of 1 mg/mL, and 22 μL of the OmpF-EIII protein solution at a concentration of 0.1 mg/mL were used, respectively.

### 4.4. Animals and Immunization

To select the dose of the antigen and the immunization schedule, the recombinant chimeric protein OmpF-EIII was injected subcutaneously in the thigh of animals at a dose of 0.02, 0.2, 2, 20, or 40 μg/mouse in a volume of 100 μL of 1× PBS containing 0.125% *n*-octylglucoside. Mice of the control group were injected only with 1× PBS containing 0.125% *n*-octylglucoside. Three immunization regimens were used: One immunization on the 1st day, two immunizations on the 1st and the 7th days, as well as two immunizations on the 1st and the 14th days. The experiment was terminated 28 days after the first immunization.

Immunization was carried out according to the established protocol [14]. Adult BALB/c mice (males) weighing 18–20 g were obtained from the ‘Pushchino’ nursery of laboratory animals at the branch of the Institute of Bioorganic Chemistry, Russian Academy of Sciences (RAS). Standard conditions with unlimited access to food and water were used to maintain these animals in a vivarium of the Pacific Institute of Bioorganic Chemistry, Far Eastern Branch (FEB) of RAS. The provisions of Directive N 2010/63/EC of the European Parliament and of the Council of the European Union “On protection of animals used for scientific purposes” were used to conduct all the experiments with animals. This study (Project identification code 0117) was approved on 16 January 2017, by the Local Ethics Committee of the Pacific Institute of Bioorganic Chemistry, FEB RAS.

The mice were divided into 5 experimental groups (10 animals in each): (1) Mice immunized with individual OmpF-EIII; (2–5) mice immunized with OmpF-EIII incorporated in TI-complexes based on MGDG from *L. japonica*, *S. pallidum*, *U. lactuca*, or *Z. marina*, respectively. Mice of the control group were injected with PBS containing 0.125% *n*-octylglucoside. Animals were injected twice subcutaneously in the thigh, applying a dose of 20 μg of OmpF-EIII per mouse, and a dose of the TI-complex containing 10 μg of CDA per mouse at an interval of 14 or 7 days. The experiment was terminated 28 days after the first immunization.

### 4.5. Enzyme-Linked Immunosorbent Assay (ELISA)

The levels of the anti-OmpF-EIII and anti-EIII antibodies in the mouse blood serum were estimated by ELISA, applying anti-mouse IgG labeled with peroxidase (N.F. Gamaleya National Research Center of Epidemiology and Microbiology, Ministry of Health of Russian Federation), as described in [11]. The blood sera of animals injected with PBS containing 0.125% *n*-octylglucoside served as a control. Sensitization of solid surface was carried out by the introduction of the OmpF-EIII or EIII solution into the wells (1 μg of the protein per well) of a 96-well microtiter plate (GosNIIMedPolimer, St. Petersburg, Russia). Absorption of the antibody samples at a wavelength of 450 nm was estimated using an Elx808IU microplate photometer (Biotek Instr., Winooski, WT, USA). To this end, 3,3′,5,5′-tetramethylbenzidine (BD, Bedford, MA, USA) was used as a chromogen. Each experiment was repeated 10 times, and the corresponding results were expressed as means ± SE. Parametric analysis using Student’s *t*-test was utilized to estimate the differences between these means, with *p* < 0.05 being considered as statistically significant.

### 4.6. Protective Activity against TBEV

Protective activity was studied in the vivarium of the G.P. Somov Research Institute of Epidemiology and Microbiology, Vladivostok, Russia. The sanitary and epidemiological rules of the Russian Federation SR 1.3.3118-13 of 28 November 2013, were followed in animal experiments that were conducted according to the European Convention for the Protection of Vertebrate Animals used for Experimental and Other Scientific Purposes (Strasbourg, 18 March 1986). Three groups (10 animals in each) of BALB/c mice (males) with a weight of 18–20 g obtained from the ‘Pushchino’ nursery of laboratory animals, a branch of the Institute of Bioorganic Chemistry, RAS, were immunized twice subcutaneously according to the procedure described before (Section 4.5): (1) PBS containing 0.125% *n*-octylglucoside (control); (2) mice immunized with individual OmpF-EIII; (3) mice immunized with OmpF-EIII incorporated in the TI-complex based on MGDG from *S. pallidum*. Experiments were replicated three times.

Two weeks after the second immunization, the mice were subcutaneously infected with 0.2 mL of TBEV strain Dal’negorsk isolated from the brain of a deceased patient with TBE, and previously described as a highly virulent Sofin-like strain of the Far-Eastern TBEV subtype (Number in GenBank FJ402886) [22,23]. Then, the survival of mice was monitored daily for 21 days. To determine the infectious dose of the TBEV, preliminary titration was performed by subcutaneous infection of mice with 0.2 mL of TBEV. The virus was used in a dose of 1–1.2 lg LD50, which induced death of 80–90% of the animals in the experiment. The Fisher Exact test was used to compare the protective activity in mice groups with the control. *p* < 0.05 was considered to indicate a statistically significant result.

### 4.7. Computational Analysis of the Intrinsic Disorder Predisposition of the Chimeric OmpF-EIII Protein

Commonly used predictors of intrinsic disorder, PONDR^®^ FIT [24], PONDR^®^ VLXT [25], PONDR^®^ VSL2 [26], MFDp [27], IUPred_short, and IUPred_long [28], were utilized in the analysis of the peculiarities of intrinsic disorder distribution within the amino acid sequence of the chimeric OmpF-EIII protein, and the mature tick-borne encephalitis virus E protein. Selection of these predictors was based on their ability to recognize different peculiarities of intrinsic disorder. PONDR^®^ VSL2B was selected for its relatively high accuracy [29,30,31]. PONDR^®^ VLXT was chosen for its high sensitivity to local sequence peculiarities that can be used for identifying disorder-based interaction sites [32]. Selection of the consensus predictor MFDp was based on its ability to generate disorder and secondary structure predictions, sequence profiles, and predictions of backbone dihedral torsion angles, B-factors, globular domains, and solvent accessibility [27]. A metapredictor PONDR-FIT [24] was selected for its moderately superior accuracy over its component predictors, PONDR^®^ VLXT [26,32], PONDR^®^ VSL2 [26], PONDR^®^ VL3 [33], FoldIndex [34], IUPred [28], and TopIDP [35]. Finally, selection of IUPred was based on the ability of this predictor to recognize protein intrinsic disorder using the estimated pairwise energy content [28,36]. Furthermore, the mean disorder propensity for these proteins based on the averaging of disorder profiles of individual predictors was also analyzed, since this approach is known to show increased predictive performance [24,31,37,38]. In these analyses, residues and regions are considered disordered or flexible if their predicted disorder scores are above 0.5, and between 0.2 and 0.5, respectively.

## Abbreviations

CDA	Cucumarioside A_2_-2
ELISA	Enzyme-linked immunosorbent assay
MGDG	Monogalactosyldiacylglycerol
PBS	Phosphate-buffered saline
TI-complex	Tubular immunostimulating complex
